# Intramuscular Hemangioma in Lip Treated with Sclerotherapy and Surgery

**DOI:** 10.1155/2011/302451

**Published:** 2011-07-06

**Authors:** Vanessa A. Silva, Nádia L. Lima, Ana Terezinha M. Mesquita, Esmeralda Maria da Silveira, Flaviana D. Verli, João Luiz de Miranda, Cássio Roberto R. Santos, Sandra A. Marinho

**Affiliations:** ^1^School of Dentistry, Federal University of Jequitinhonha and Mucuri Valleys (UFVJM), 39100-000 Diamantina, MG, Brazil; ^2^Laboratory of Pathology, Department of Basic Sciences, Federal University of Jequitinhonha and Mucuri Valleys (UFVJM), 39100-000 Diamantina, MG, Brazil; ^3^Stomathology Clinics, School of Dentistry, Federal University of Jequitinhonha and Mucuri Valleys (UFVJM), 39100-000 Diamantina, MG, Brazil; ^4^Post Graduate Program in Dentistry, Federal University of Jequitinhonha and Mucuri Valleys (UFVJM), Rua da Glória 187, 39100-000 Diamantina, MG, Brazil

## Abstract

Intramuscular hemangioma is a relatively rare, benign tumor of vascular origin, accounting for less than 1% of all hemangiomas. This paper reports a case of a 48-year-old female patient with intramuscular hemangioma in the upper lip, treated with sclerotherapy and subsequent complementary surgery.

## 1. Introduction

Hemangiomas are benign vascular alterations characterized by the proliferation of blood vessels, commonly occurring in subcutaneous and submucous tissue [1–6]. When located superficially, a hemangioma is easy to diagnosis. However, in the relatively uncommon occurrence deeper in the tissue, hemangioma is often difficult to diagnose [7–11]. 

When the proliferation of blood vessels is found between skeletal muscle fibers, such tumors are denominated intramuscular hemangioma (IMH), which accounts for less than 1% of all hemangiomas [5]. IMHs occur more frequently on the trunk and extremities as well as in the head and neck region, which account for 10 to 20% of all IMHs [3–6]. The diagnosis of IMH is determined through biopsy following the sudden growth of the tumor [3, 6]. The predominant complaint is the presence of a frequently painful, slow-growing nodule with a negative esthetic effect [3]. IMHs have no predilection for either gender [5].

Given the rarity, deep location, and variable clinical presentation of these tumors [5], the aim of the present study was to describe a clinical case of IMH in the upper lip treated with intratumor administration of monoethanolamine oleate and subsequent surgery.

## 2. Case Presentation

**Figure 1:**
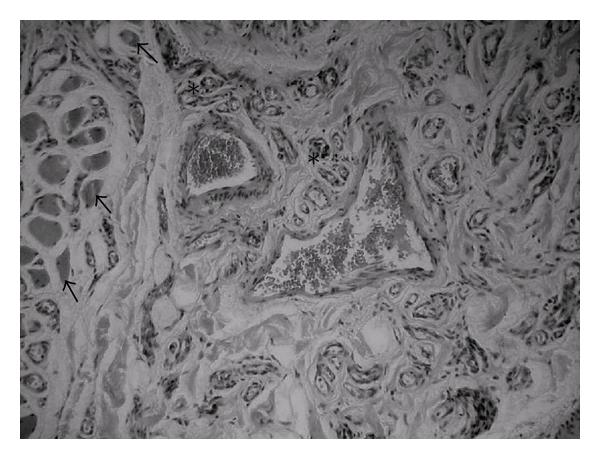
Capillaries (stars) invading muscle tissue (arrows; 200x, HE).

**Figure 2:**
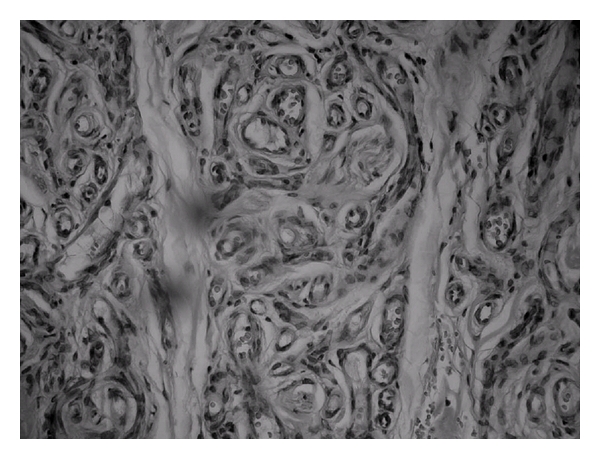
Proliferation of capillaries (400x, HE).

**Figure 3:**
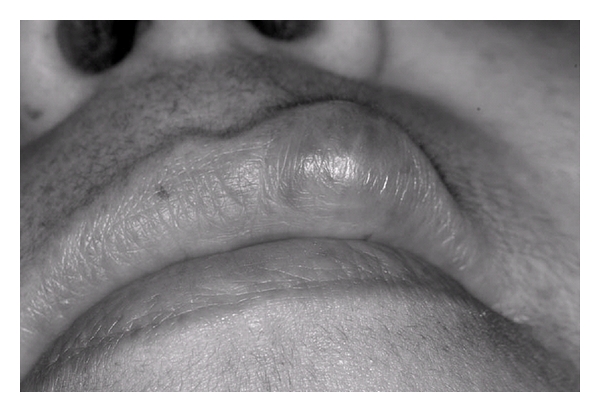
Clinical aspect of submucous nodule following sclerotherapy.

**Figure 4:**
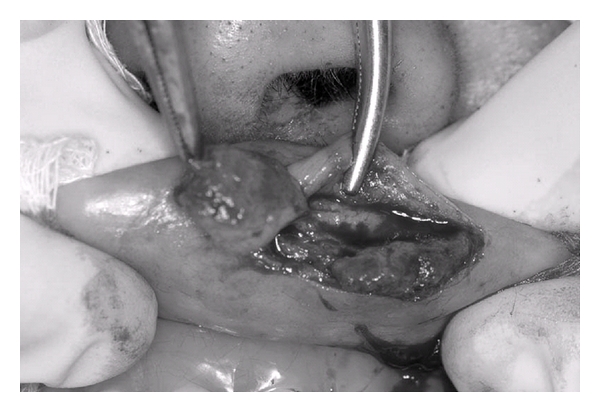
Excisional biopsy.

**Figure 5:**
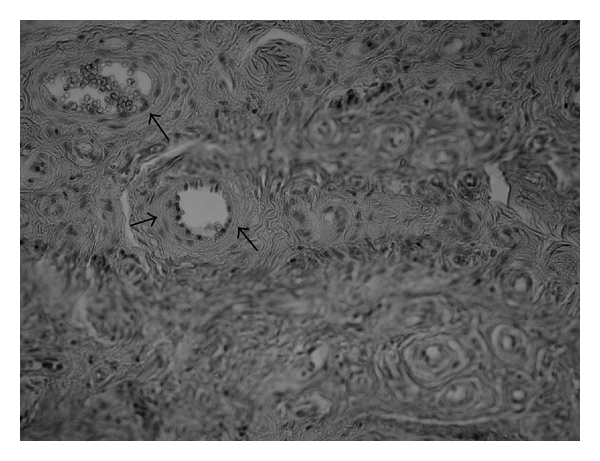
Proliferation of capillaries with hyaline perivascular material (arrows; 400x, HE).

A 48-year-old female patient came to the stomatology service complaining of swelling in the upper lip. The patient history revealed that the lesion was asymptomatic, with evolution of approximately four years without treatment. The extraoral exam revealed asymmetry between the upper and lower lips. The intraoral exam revealed a submucous nodule coated with normal mucous, with firm consistency to the touch as well as clear limits and mobility. The initial diagnostic hypotheses were fibroma, pleomorphic adenoma, and neurilemmoma. Following incisional biopsy, the histopathologic exam revealed the proliferation of vascular capillaries invading the muscle tissue (Figure 1), leading to the diagnosis of capillary hemangioma (Figure 2). The treatment selected was a series of applications of 5% Ethamolin (Zest farmacêutica, Rio de janeiro, RJ, Brazil) diluted in distilled water (1 : 1) for a final concentration of 2.5%. After three applications, the patient did not return to the clinic for three years after the initial biopsy (Figure 3). As there was partial total regression of the lesion, excisional biopsy of the submucous nodule was performed (Figure 4). The histopathologic exam revealed the proliferation of vascular capillaries, with the presence of a perivascular hyaline material in the muscle tissue (Figure 5), leading to the diagnosis of intramuscular hemangioma (IMH). The patient has since been in followup and has shown no signs of recurrence or any other changes associated to the injury.

## 3. Discussion

Clinically, the differential diagnosis of submucous hemangioma includes cyst, mucocele, and Kaposi's sarcoma. The vitropressure maneuver makes a hemangioma acquire a pale coloration and reduce in size due to the emptying of its blood vessels [12–14]. This procedure was not carried out in the case reported here, as the lesion was not located superficially and did not exhibit the typical purplish color of a hemangioma. Thus, the hypotheses initially raised were fibroma, pleomorphic adenoma, and neurilemmoma, rather than hemangioma [15]. 

The slow growth with an evolution of four years indicated a likely benign nature of the tumor. Kanaya et al. [5] reported the presence of painful tumefaction in the right cheek of a 14-year-old patient since the child was three years of age, with a gradual increase in size and the diagnosis of IMH of the masseter muscle.

In the present case, the patient had not undergone any type of treatment since the perception of the first indications of the nodule and only sought treatment for esthetic reasons. Affected patients generally seek treatment due to esthetic or functional problems [16]. Surgical excision resulted in the functional and esthetic resolution of six cases reported by Ranero-Juárez et al. [16]. This is the first treatment choice in patients with systemic alterations, significant deformities, or refractory lesions stemming from other treatments. 

In the case reported here, the tumor was located in the orbicular muscle of the upper lip, with a nodular appearance and absence of pain symptoms. However, such tumors may be accompanied by pain [8, 17–19]. According to Nam and Hwang [6], the occurrence of IMH in an oral orbicular muscle is rare. In the head and neck region, IMH is mainly found in the masseter, trapezium, periorbital, sternocleidomastoid, or oral orbicular muscles. The involvement of adjacent structures may include the parotid gland, pterygoid muscles, or infratemporal fossa [3]. Despite the broad anatomic distribution, however, IMH most commonly occurs in the extremities [8, 17–19].

In the present case, the initial conduct was incisional biopsy of the tumor for diagnostic purposes. Bleeding was easily controlled during the procedure. The histopathologic analysis revealed the proliferation of capillaries invading the muscle tissue. There was also the proliferation of endothelial cells due to increased mitotic activity, with the accumulation of thin-walled capillaries separated by sparse conjunctive tissue stroma. The diagnosis was determined as capillary hemangioma [14, 20, 21]. 

The treatment selected was sclerotherapy. However, before choosing the adequate type of treatment for a hemangioma, a number of characteristics should be considered, such as duration, size, location and number of tumors, patient age, and the hemodynamics of the tumor. Moreover, the viability of the intended technique must also be assessed [22].

Sclerotherapy with monoethanolamine oleate was chosen due to its safety and ease of application and the fact that the treatment could be repeated without risk in the case of recurrence of the nodule [23]. Through an intratumor injection, monoethanolamine oleate either completely or partially reduces the size of the tumor so that surgical excision can be performed with a greater degree of safety. Causing the safe involution of the tumor through a nonsurgical procedure and favoring both the postoperative period and patient esthetics are important factors to be considered in the decision as to what treatment to employ [24].

Monoethanolamine oleate initially irritates the venous or capillary epithelium, thereby producing an extra-vascular inflammatory response, which results in fibrosis and the occlusion of the blood vessels [25]. This agent also causes tissue necrosis and the formation of local thromboses, observed 24 hours following application [21, 25, 26]. The patient in the present case only reported slight discomfort during and following the administration of the sclerosing agent. The postoperative pain may be explained by the induction of a local inflammatory response due to the intratumor injection of the agent. Pain symptoms, however, should not extend for more than three days [21, 26].

It should be borne in mind that an injection of a volume greater than the recommended amount can cause extensive tissue necrosis and trigger an anaphylactic reaction in patients who are sensitive to the drug [27]. Moreover, the application should always be carried out in the center and deepest portion of the tumor in order to avoid tissue necrosis. This care was taken during the application of the sclerosing agent in the present case. Moreover, the substance was diluted, resulting in a lesser concentration [21] in an effort to minimize tissue necrosis.

Sclerotherapy and surgery are the most often employed techniques in the treatment of hemangiomas of the mouth [14]. Surgery can be employed in the form of embolization, selective arterial ligation, or simple exeresis with or without plastic reconstruction [22, 28, 29]. According to Odabasi et al. [3], the most indicated treatment for IMH is surgical removal with a margin of safety due to the infiltrative nature of the tumor; the nonaffected portion of the muscle beyond the limits of the tumor should also be removed in order to avoid local recurrence.

After three sessions of sclerotherapy, the patient did not appear for followup and only returned three years after the biopsy. Due to the residual presence of the tumor, the decision was made for the surgical removal of the submucous nodule, establishing the diagnosis of capillary IMH, with the proliferation of capillaries and the presence of hyaline perivascular material intercalated in the muscle tissue. The surgery was used as a complementary treatment to sclerotherapy. However, if the patient had shown up for the remaining scheduled sessions for the administration of the sclerosing agent, there may have been no need for surgery.

The patient is currently in followup, with no clinical signs of recurrence or any other condition associated to the tumor. Although local recurrence may occur in more than 50% of cases, it has no correlation with the type of predominant vessel or anatomic location of the tumor and there are no reports of malignant transformation in cases of incomplete removal [7, 30]. Kanaya et al. [5] reported a case of recurrence nine months following conservative surgery of an IMH in the masseter muscle. In an additional surgical approach, the authors removed a portion of the buccal branch of the facial nerve in order to achieve complete resection of the tumor. According to Odabasi et al. [3], the intraoral approach in such cases offers limited exposure of the branches of the facial nerve, which may result in nerve injury and postoperative paralysis; therefore, an extraoral approach is required. As the tumor in the case reported here was well circumscribed and well delimited due to the fibrosis in the region caused by sclerotherapy, there were no difficulties in the execution of its complete excision.

## 4. Conclusion

It is important for dentists to know that diascopy is of considerable assistance to the correct diagnosis of superficial vascular lesions. However, when such tumors are located more deeply in the tissue, a biopsy is essential to the diagnosis. Moreover, one must take into consideration the correct indication of sclerosing agents for treatment.
